# RP-DAD-HPLC Method for Quantitative Analysis of Clofazimine and Pyrazinamide for Inclusion in Fixed-Dose Combination Topical Drug Delivery System

**DOI:** 10.3390/mps9010016

**Published:** 2026-01-21

**Authors:** Marius Brits, Francelle Bouwer, Joe M. Viljoen

**Affiliations:** Faculty of Health Sciences, Centre of Excellence for Pharmaceutical Sciences (Pharmacen^TM^), North-West University, Building G16, 11 Hoffman Street, Potchefstroom 2520, South Africa; francellebouwer@gmail.com (F.B.); joe.viljoen@nwu.ac.za (J.M.V.)

**Keywords:** clofazimine, pyrazinamide, tuberculosis, high-performance liquid chromatography (HPLC), topical drug delivery system

## Abstract

Reversed-phase high-performance liquid chromatography (RP-HPLC) remains one of the most widely applied analytical techniques in the development and quality control testing of finished pharmaceutical products. The combination of gradient chromatographic methods with diode-array detection (DAD) enhances selectivity, ensuring accuracy and reliability when testing drugs with diverse chemical properties in a single dosage form (i.e., fixed-dose combination (FDC) products). In this study, an RP-DAD-HPLC method was developed for the quantitative analysis of clofazimine (CFZ) and pyrazinamide (PZA) for inclusion in an FDC topical drug delivery system. Chromatographic separation was achieved using a C18 column (4.6 mm × 150 mm, 5 µm particle size) with gradient elution at 1 mL/min, employing 0.1% aqueous formic acid and acetonitrile (mobile phases). PZA and CFZ were detected at 254 nm and 284 nm, respectively. The method was validated in accordance with ICH Q2 guidelines, assessing specificity (considering interference from solvents, product matrix, and degradation products), linearity (7.8–500.0 µg/mL, r^2^ = 0.9999), system repeatability (%RSD ≤ 2.7%), and intermediate precision (25–500 µg/mL, %RSD ≤ 0.85%). Method robustness was evaluated using a three-level Box–Behnken design (BBD) with response surface methodology (RSM) to assess the effects of variations in detection wavelength, mobile phase flow rate, and column temperature.

## 1. Introduction

In 1826, Théophile Laennec, the inventor of the stethoscope [1], reported the first case of an extrapulmonary tuberculosis (EPTB) variant known as true cutaneous tuberculosis (CTB), a rare skin infection primarily caused by *Mycobacterium tuberculosis*, though other *Mycobacterium* species may also be implicated. CTB remains difficult to diagnose due to its diverse and often deceptive clinical manifestations, which frequently mimic other dermatological conditions. As a result, misdiagnosis is common, treatment is delayed, and tissue damage may progress [2]. Despite increasing research attention, CTB still lacks a universally accepted definition or diagnostic standard. The current classification systems attempt to integrate pathogenesis, clinical morphology, and histopathological findings, without converging on a single standardized framework [3].

Although the World Health Organization (WHO) has no CTB-specific guideline, the global tuberculosis (TB) policy documents do recognize the skin as one of the possible sites of extrapulmonary involvement [4]. Current WHO operational handbooks and treatment regimens address EPTB broadly, yet disease-specific criteria for CTB remain undeveloped [4]. Thus, CTB continues to be discussed mainly in scientific literature rather than in global policy [5].

TB is generally preventable and curable [5], yet WHO statistics show that in 2023 it again became the leading cause of death worldwide from a single infectious agent, surpassing COVID-19 [5]. TB caused nearly twice as many deaths as the human immunodeficiency virus (HIV) and acquired immune deficiency syndrome (AIDS) in the same year, and its global incidence has continued to rise, with over 10 million new cases annually [5]. Although CTB accounts for only 1.5–3% of all EPTB cases [2,6,7], increasing multidrug-resistant TB has contributed to its growing clinical relevance [8]. The global TB burden translates to an estimated 150,000 CTB cases annually. In high-TB-burden regions, CTB accounts for roughly 2% of dermatology outpatient visits, and about one-third of affected patients also present with systemic TB, underscoring the importance of timely recognition by dermatologists [2].

CTB management generally follows WHO regimens for drug-susceptible TB, as no CTB-specific regimens exist. Standard therapy consists of a two-month intensive phase using isoniazid, rifampicin, pyrazinamide (PZA), and ethambutol, followed by at least four months of treatment with isoniazid and rifampicin [2,9]. For multidrug-resistant or rifampicin-resistant TB, the WHO recommends all-oral regimens incorporating bedaquiline, pretomanid, linezolid, and moxifloxacin. In an instance where fluoroquinolone resistance is present, moxifloxacin is excluded [10]. National TB programs, such as those in South Africa, adopt these protocols considering drug availability, HIV co-infection, and disease severity [11]. Treatment adherence remains a significant challenge due to long treatment durations and severe adverse effects experienced [5,12].

Topical drug delivery offers a promising alternative for CTB treatment, as it is non-invasive, targets the site of infection directly, and may reduce systemic side effects [13,14].

Recent research has focused on fixed-dose drug combinations (FDCs), which exploit drug synergy to enhance therapeutic outcomes, reduce dosing frequency, and lower drug resistance risks [15,16,17]. However, FDC development requires careful dosing, compatible pharmacokinetics, and avoidance of drug-induced toxicity [16].

In another study, we explored the feasibility of developing a topical drug delivery system for CTB treatment, employing FDC therapy incorporating clofazimine (CFZ) and pyrazinamide (PZA). Oral CFZ–PZA combinations have been shown to significantly reduce the required CFZ dose from 25 mg/kg to 6.25 mg/kg [18] supporting their synergistic potential [19,20]. CFZ is effective against multi-drug-resistant TB strains, but its use is limited owing to its dose-dependent skin discoloration [19,20,21,22,23], severe gastrointestinal side effects, ocular deposition, and dark-brown fingernail discoloration. These side effects arise from CFZ’s highly lipophilic nature (Log P of 7.66) [24], which causes prolonged accumulation in adipose tissue for up to 70 days [25,26]. Consequently, incorporating a reduced quantity of CFZ into a topical drug delivery system would be beneficial, as it could decrease systemic adverse effects by limiting systemic absorption [25,26,27].

However, CFZ’s poor aqueous solubility complicates formulation development [27,28]. Furthermore, PZA, a nicotinamide analog used in combination with drugs such as rifampicin and isoniazid during the initial treatment phase [29,30,31], is a relatively hydrophilic drug (Log P of −0.71) [24]. Although its physicochemical properties are not ideal for dermal drug diffusion, PZA remains essential due to its primarily bacteriostatic nature, with bactericidal effects against *Mycobacterium tuberculosis* in acidic environments (pH ≤ 5.6) [32], and it shortens the treatment duration [33]. Orally, PZA is associated with gastrointestinal discomfort, hepatotoxicity, nausea, and vomiting [34]. Therefore, an alternative administration route, such as dermal delivery, could provide substantial benefits by directly targeting the infection site, lowering the CFZ concentration, reducing individually caused side effects, and ultimately improving patient compliance.

In developing an FDC combining a highly lipophilic drug (i.e., CFZ) with a highly hydrophilic drug (i.e., PZA), solubilizing both drugs is challenging. Lipid-based formulations, such as spontaneously forming emulsions, are commonly used as carriers for poorly water-soluble drugs [35,36,37,38]. The manufacturing process allows for the incorporation of existing anti-tubercular drugs into an FDC within a topical formulation. In this system, the highly lipophilic CFZ is dissolved in the oil phase and the highly hydrophilic PZA in the aqueous phase. As a result, both CFZ and PZA are dissolved and available for absorption within the topical drug delivery system, thereby overcoming solubilization challenges and improving drug delivery [39].

To co-deliver the highly lipophilic CFZ and hydrophilic PZA, topical microemulsion formulations using different oil phases were manufactured. Olive oil (OLV) was chosen as the “base” oil, and was also combined with 1.5% (*v*/*v*) of different essential oils, namely eucalyptus oil (EUC), peppermint oil (PPO), and tea tree oil (TTO). The use of plant-based oils in pharmaceutical formulations has become increasingly prevalent, as they have been shown to enhance skin penetration by temporarily altering the barrier function of the stratum corneum [40]. Furthermore, these oils can promote enhanced dermal drug penetration by creating an occlusive effect that reduces transepidermal water loss and improves skin hydration [41]. Additionally, to ensure proper solubilization of CFZ (0.7% *w*/*v*) and PZA (2% *w*/*v*) and to form stable microemulsions, a surfactant/cosurfactant combination comprising equal volumes of Span^®^83 (surfactant) and Tween^®^60 (cosurfactant) was included.

**Figure 1:**
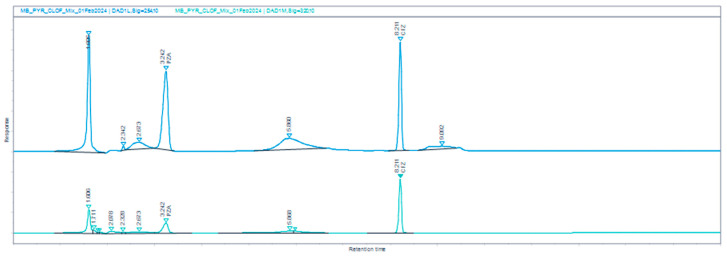
Chromatograms obtained for a standard solution containing ~250 µg/mL CFZ and PZA in methanol recorded at two wavelengths: 320 nm (cyan) and 254 nm (dark blue), respectively.

An existing HPLC method [42] was initially considered for quantifying PZA and CFZ in solubility studies (in water, in OLV, and in the mixtures of OLV with 1.5% (*v*/*v*) EUC, PPO, or TTO), emulsions, and in vitro drug release studies. However, injections of CFZ-PZA standards produced unusable chromatograms (Figure 1) showing peak deformities, unknown peaks, and baseline drifts. Consequently, this method could not be successfully transferred to our laboratory.

As reported in the literature, a common reason for HPLC method non-transferability is its lack of robustness [43]. Unfortunately, the cited article [42] did not provide any optimization or robustness data. This necessitated the development and validation of a new HPLC method, which is presented in this article.

The objective of this study was to develop and validate a rapid and reliable HPLC method capable of accurately quantifying the solubility of CFZ and PZA, assaying the amounts of each drug in the topical FDC emulsions, and determining the concentration of the FDC on, in, and through the skin following dermal drug delivery from the various emulsions tested. Method development and validation were performed in accordance with the International Council for Harmonization (ICH) and Good Manufacturing Practice (GMP) requirements [44] and presented in the following sections. The detailed experimental setup and results from using the developed method for solubility studies, assays, and transdermal diffusion studies are presented in a companion publication entitled: “Quality-by-Design Optimization of Self-Emulsifying CFZ/PZA Emulsions and Comparative Diffusion Across Strat-M^®^ and Human Skin”.

## 2. Experimental Design

### 2.1. Materials

CFZ was acquired from Cipla (Cape Town, South Africa), and PZA was obtained from DB Fine Chemicals Pty Ltd. (Sandton, South Africa). The various oil phases used (i.e., OLV, EUC, PPO, and TTO) were obtained from Scatters Oils (Johannesburg, South Africa). Hydrochloric acid, sodium chloride, and hydrogen peroxide (3%), all analytical grade, were sourced from Merck (Modderfontein, Johannesburg, South Africa). Ultra-purified water was produced using a Rephile Bioscience Ltd. Genie U12 water purification system (Boston, MA, USA).

Span^®^83 (sorbitan sesquioleate) and Tween^®^60 (polyoxyethylene sorbitan monostearate or polysorbate 60) were purchased from Sigma-Aldrich Chemistry GmbH (Steinheim, Germany). The HPLC-grade methanol and acetonitrile used were supplied by Fisher Chemical as Fisher Scientific Company L.L.C. (Pittsburgh, PA, USA). EMSURE^®^ ACS Ph. Eur. grade Formic acid (98–100%) was procured from Merck KGaA (Darmstadt, Germany).

### 2.2. Equipment

A Hitachi Chromaster HPLC system (Hitachi High-Tech Science Corporation, Tokyo, Japan) was employed in this study. The HPLC system was equipped with a Chromaster 5160 quaternary pump, a Chromaster 5280 temperature-controlled autosampler, a Chromaster 5310 column oven, and a Chromaster 5430 diode array detector with a variable wavelength detector. A Luna^®^ C18(2) 100 Å CC column (4.6 mm × 150 mm, 5 µm particle size) from Phenomenex (Torrance, CA, USA) was used. The injection volume was set to 1 µL, and the flow rate was set to 1.0 mL/min. Mobile phase A was a 0.1% aqueous formic acid solution, and mobile phase B was HPLC-grade acetonitrile. The gradient program employed during the analysis is summarized in Table 1.

### 2.3. HPLC Operating Conditions

Data acquisition and analysis were performed using OpenLab CDS PLUS software version 2.7. The chromatographic system was operated with an injection volume of 1 µL and a flow rate of 1.0 mL/min. The mobile phases consisted of 0.1% aqueous formic acid solution (mobile phase A) and HPLC-grade acetonitrile (mobile phase B). The gradient elution program applied during the analysis is summarized in Table 1.

## 3. Procedures

### 3.1. Preparation of Stock and Standard Solutions

Stock standard solutions containing approximately 500.0 µg/mL of PZA and CFZ in methanol were prepared. Each stock solution was serially diluted with methanol to obtain solutions containing approximately 400.0, 250.0, 125.0, 62.5, 15.6, and 7.8 µg/mL of PZA and CFZ. These solutions served to validate the method, as described in the following sections.

### 3.2. Chromatographic Properties of the Eluted Peaks

The symmetry of the eluted peaks was assessed by calculating the tailing factor (T_F_) using Equation (1), as described in the available literature [45]:

(1) $$T_{F}\;=\;\frac{W_{0.05}}{2d}$$

where: (W_0.05_) represents the width of the peak at one-twentieth of the total peak height, and (d) denotes the distance between the perpendicular line dropped from the peak maximum and the leading edge of the peak at one-twentieth of the total peak height.

The resolution (R_S_) between the PZA and CFZ peaks was calculated using Equation (2) [45]:

(2) $$R_{s}\;=\;\frac{1.18\;\times \;\left(t_{R2}\;-\;t_{R1}\right)}{W_{h1}+\;W_{h2}}$$

where: (R_s_) indicates the R_S_ between the peaks of PZA (Peak 1) and CFZ (Peak 2), (t_R1_) and (t_R2_) are the retention times of the PZA and CFZ peaks, respectively, and (W_h1_) and (W_h2_) represent the widths at half-height for the PZA and CFZ peaks, individually.

### 3.3. Method Validation Procedure

Method validation was performed by assessing the following parameters: system repeatability, specificity, linearity and range, limit of detection, limit of quantitation, accuracy, intermediate precision, and robustness.

#### 3.3.1. System Repeatability

System repeatability was evaluated by injecting a standard solution containing approximately 250 µg/mL of PZA and CFZ in methanol six times and calculating the relative standard deviation (%RSD) values of the instrument responses, namely the area under the curve (AUC) and the retention time (T_R_) for both drugs. In accordance with the United States Pharmacopoeia (USP) requirements, the %RSD for system repeatability must not exceed 3.3% [45].

#### 3.3.2. Specificity

Method specificity was assessed in accordance with the ICH Q2(R2) guidelines, considering chromatographic R_S_ and peak purity [44]. Specificity studies determine the ability of the analytical method to unambiguously detect and quantify analytes in the presence of other components, such as solvents, formulation matrices, and potential analyte degradation products.

To demonstrate selectivity, the solvents (i.e., water, OLV, and OLV essential oil mixtures consisting of OLV+ 1.5% (*v*/*v*) EUC; OLV + 1.5% (*v*/*v*) PPO; and OLV + 1.5% (*v*/*v*) TTO), as well as placebos of the various PZA-CFZ FDC topical emulsions, were analyzed using methanol as the solvent to detect potential interference with the R_T_s of the PZA and CFZ peaks.

Forced degradation studies were conducted under acidic (0.1 N HCl), basic (0.1 N NaOH), and oxidative (3% (*v*/*v*) H_2_O_2_) conditions at an elevated temperature of 60 °C to detect potential interference from degradation products. Individual standard solutions of PZA and CFZ were prepared by dissolving 100 mg of the respective reference standard in 50 mL of methanol and diluting it to a final volume of 100 mL with the indicated stressor solutions. The resulting solutions were incubated at 60 °C for 3 h, after which aliquots were diluted with methanol to an approximate final concentration of 500 µg/mL before HPLC analysis.

#### 3.3.3. Linearity, Range, Limit of Detection, and Limit of Quantitation

Linearity and range of the analytical method were established by preparing serial dilutions from the stock solutions described in Section 3.1. Calibration curves were constructed using the six calibration points, which ranged from 7.8 to 500.0 µg/mL. The limit of detection (LOD) and limit of quantitation (LOQ) were determined as described in the ICH Q2 guideline [44].

#### 3.3.4. Accuracy

Accuracy and precision of the analytical method were evaluated by preparing spiked solutions at three concentration levels (25, 250, and 500 µg/mL) and calculating the recovery of PZA and CFZ using Equation (3):

(3) $$\%\;\mathrm{Recovery}\;=\;\frac{\mathrm{Experimental}\;\mathrm{concentration}}{\mathrm{Theoretical}\;\mathrm{concentration}}\;\times 100$$

#### 3.3.5. Intermediate Precision

Intermediate precision was investigated by analyzing spiked solutions at three concentration levels (25, 250, and 500 µg/mL) prepared on three different days (Day 1, Day 2, and Day 3) as part of the accuracy study. The % recovery values of PZA and CFZ were determined from six replicate injections, and the %RSD values for the individual and combined data sets were calculated.

#### 3.3.6. Robustness

Method robustness was examined by introducing deliberate changes to the system parameters, including detection wavelength, mobile phase flow rate, and column temperature. A standard solution containing approximately 250 µg/mL of PZA and CFZ in methanol was used for all the experimental runs. The effects of these variations were evaluated using a three-level Box–Behnken design (BBD) and response surface methodology (RSM) approach commonly applied within Quality-by-Design (QbD) principles, which has gained popularity in assessing method robustness of HPLC methods [46,47,48,49]. The benefits of using RSM include the fact that it allows for a more efficient experimental design (requiring fewer runs than a full factorial design), and it evaluates interaction effects and aligns with Analytical QbD (AQbD) approaches commonly required in regulatory submissions.

The BBD incorporated three independent and six dependent variables. In this study, the independent variables included the wavelength of detection (*x*_1_, nm), flow rate (*x*_2_, mL/min), and column temperature (*x*_3_, °C). The dependent factors included the R_T_ (*y*_1_, min) of PZA or CFZ, the T_F_ (*y*_2_) of PZA or CFZ, and the R_S_ factor (*y*_3_) observed under the respective conditions. Table 2 summarizes the levels of the independent variables, while Table 4 outlines the experimental design used in this study and the results obtained.

The effects of the independent variables on the responses were analyzed using Statistica software (version 14.0.0.15; TIBCO^®^, San Ramon, CA, USA), which generated the polynomial equations describing the relationships among variables. The model quality was assessed using the correlation coefficient (r^2^), adjusted correlation coefficient (Adj. r^2^), and the analysis of variance (F-test). Acceptable model criteria include r^2^ > 0.900, a difference between r^2^ and Adj. r^2^ ≤ 0.2, and a *p*-value < 0.05 for the F-test. Contour plots of the tested responses were constructed from the fitted polynomial models to visualize changes in the response surfaces under varying analytical conditions.

## 4. Results and Discussion

### 4.1. Chromatographic Analysis

As illustrated in Figure 1, the analytical method proposed by van Staden et al. [42] was unfortunately not inter-laboratory transferable. The method was subsequently adjusted, retaining the use of 0.1% aqueous formic acid and acetonitrile as mobile phases, since this combination is commonly employed in HPLC gradient method development and provides adequate solubility for both analytes [50]. Formic acid establishes an acidic environment that promotes the ionization of weakly basic drugs such as PZA, thereby improving the shapes by suppressing the ionization of silanol groups in the stationary phase. Furthermore, acetonitrile serves as an ideal organic modifier to facilitate the separation between the hydrophilic (PZA) and lipophilic (CFZ) drugs.

Upon reviewing the gradient conditions of the published method, rapid changes in the mobile phase composition were identified as a potential source for the observed problems. Consequently, the gradient was modified as described in the Methodology section (Table 1).

In the study by Van Staden et al. [42] the detection wavelengths of 254 nm and 320 nm were used for isoniazid/PZA and rifampicin/CFZ, respectively. Considering the UV_max_ values reported in the literature for the two drugs (UV_max_ − PZA = 269 nm and UV_max_ − CFZ = 284 nm) [51], the detection wavelength for PZA was maintained at 254 nm, as the peak response obtained was sufficient to prevent detector overload at higher concentrations. However, the detection wavelength for CFZ was adjusted to 284 nm to enhance detector sensitivity.

**Figure 2:**
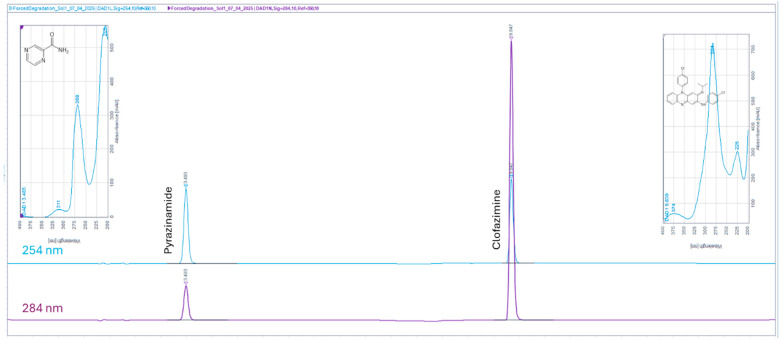
Chromatograms obtained for a standard solution containing PZA and CFZ recorded at two wavelengths—254 nm (blue) and 284 nm (purple)—individually.

The chromatogram obtained using the adapted method (Figure 2) for a standard solution containing both analytes (PZA and CFZ) showed two distinct peaks. PZA, the hydrophilic drug, eluted at approximately 3.493 min, while CFZ, the lipophilic drug, eluted at around 9.847 min.

### 4.2. Validation of the Analytical Method

The system repeatability results complied with the USP specifications, as the %RSD values of both the AUC and TR for both PZA and CFZ were below 3.3: %RSD_AUC-PZA_ = 1.0%, %RSD_TR-PZA_ = 0.1%, %RSD_AUC-CFZ_ = 1.0%, and %RSD_TR-CFZ_ = 0.4%.

The calculated T_F_s for the two peaks were 1.1 and 1.5, respectively, both of which fall within the acceptable T_F_ range of 1.0–1.5. The R_S_ between the PZA and CFZ peaks was determined to be 44.4, indicating complete baseline-to-baseline separation (discussed in Section 4.3).

As previously mentioned, the selectivity of the method was evaluated in accordance with the ICH Q2(R2) guidelines, considering chromatographic R_S_ and peak purity analysis [44]. According to ICH Q2(R2), the specificity of an HPLC method is demonstrated by the absence of interference from excipients, solvents, and potential degradation products [44].

The chromatograms of the solvents used during the solubility studies, namely water, OLV, and OLV mixtures containing 1.5% (*v*/*v*) of selected essential oils (EUC, PPO, and TTO), each diluted with methanol in a 50:50 ratio, did not display any peaks. Therefore, no interference with the PZA and CFZ peaks was detected.

**Figure 3:**
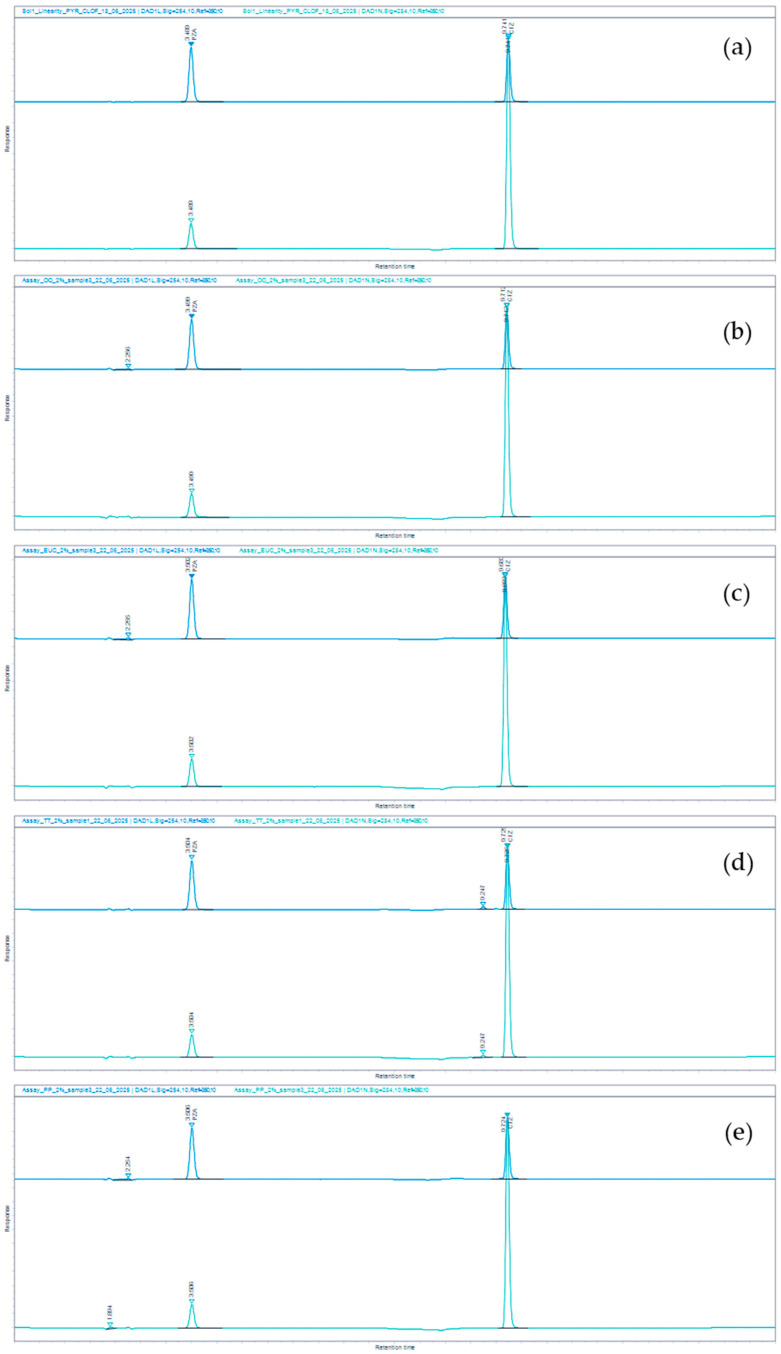
HPLC chromatograms of (**a**) CFZ-PZA reference standard solution, (**b**) CFZ-PZA microemulsion in the OLV base, (**c**) CFZ-PZA microemulsion in the OLV&EUC base, (**d**) CFZ-PZA microemulsion in the OLV&TTO base, and (**e**) CFZ-PZA microemulsion in the OLV&PPO base. Each was recorded at two wavelengths: 254 nm (dark blue) and 284 nm (cyan), respectively.

Figure 3 illustrates examples of chromatograms obtained during the assay testing of the microemulsions prepared in OLV, and the OLV mixtures containing the mentioned essential oils. These chromatograms illustrate that the excipients present in the various microemulsions did not co-elute with either drug.

**Figure 4:**
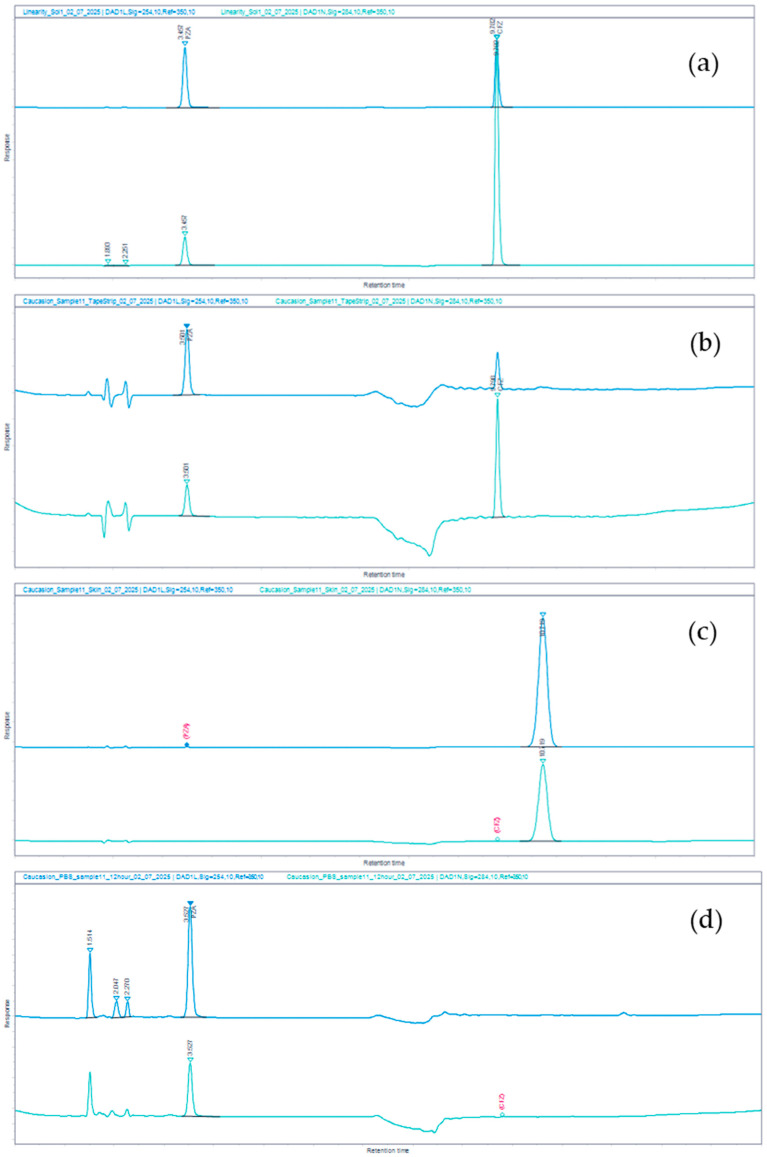
HPLC chromatograms of the samples analyzed during the transdermal diffusion studies to quantify (**a**) CFZ-PZA reference standard solution, (**b**) CFZ-PZA diffused onto the skin (stratum corneum tape striped samples), (**c**) CFZ-PZA diffused into the epidermal-dermal layer, and (**d**) CFZ-PZA diffused through the human skin into the acceptor chamber. Each was recorded at two wavelengths: 254 nm (dark blue) and 284 nm (cyan), respectively.

As mentioned earlier, in a companion publication entitled “Quality-by-Design Optimization of Self-Emulsifying CFZ/PZA Emulsions and Comparative Diffusion Across Strat-M^®^ and Human Skin”, the release of CFZ and PZA from the most optimal microemulsion (i.e., OLV&PPO base), and subsequent transport onto, into, and through human skin (Caucasian and African skin) are discussed. Figure 4 depicts examples of HPLC chromatograms of samples analyzed during the transdermal diffusion studies, showing baseline separation of PZA and CFZ from endogenous skin-derived components under the validated conditions for Caucasian skin. Similar chromatographic behavior was observed when African skin was used. The reasons for the limited or no transport of CFZ and/or PZA into the epidermal-dermal region and through the skin have been discussed in the companion publication.

The method’s selectivity toward degradation products was verified through forced degradation studies. In these studies, PZA and CFZ were exposed to acidic (0.1 N HCl), basic (0.1 N NaOH), and oxidative (3% H_2_O_2_) stressors at 60 °C for 3 h. The percentage degradation observed in PZA and CFZ under the various stress conditions is summarized in Table 3.

**Figure 5:**
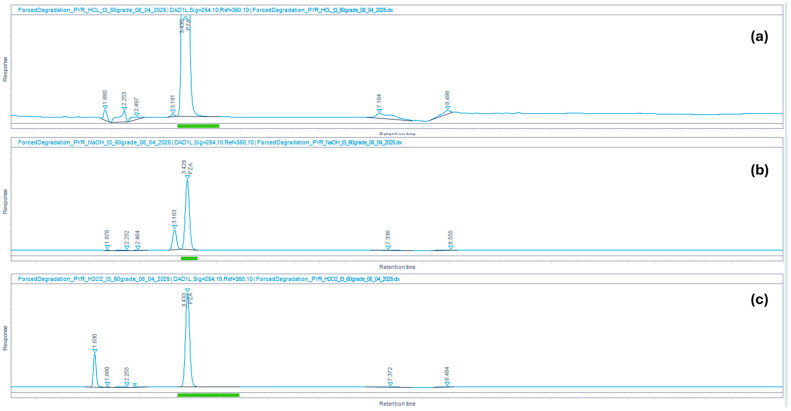
HPLC chromatograms showing PZA and its degradation products following exposure to (**a**) acidic (0.1 N HCl), (**b**) basic (0.1 N NaOH), and (**c**) oxidative (3% H_2_O_2_) stress conditions for 3 h at 60 °C.

PZA was found to be more susceptible to basic than acid stress conditions, consistent with findings reported by Lakshmi and Jacob [52]. Under acidic conditions, PZA showed only 0.4% degradation. A small peak appearing at 3.191 min in the chromatogram (Figure 5a) could be attributed to a degradation product. The R_S_ between PZA and this peak was 1.8, indicating that the two adjacent peaks are baseline-resolved (R_S_ ≥ 1.5) [45]. Under basic conditions, PZA showed approximately 26.7% degradation, with the degradation product eluting at 3.183 min (Figure 5b) and an R_S_ of 1.7 relative to the PZA peak. Exposure of PZA to oxidative stress conditions for 3 h did not result in detectable degradation. The purity (spectral match factor) for the PZA peak (Figure 5b) was 99.98%, confirming that the peak is spectrally pure and unlikely to contain co-eluting impurities or degradation products.

**Figure 6:**
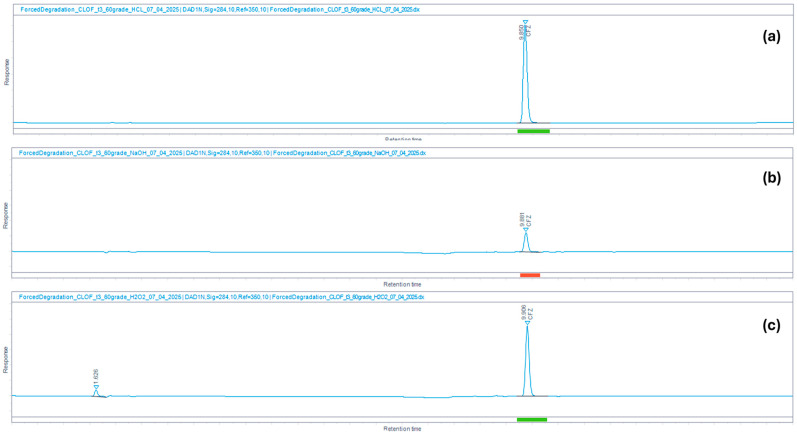
HPLC chromatograms showing CFZ and its degradation products exposed to (**a**) acidic (0.1 N HCl), (**b**) basic (0.1 N NaOH), and (**c**) oxidative (3% H_2_O_2_) stress conditions for 3 h at 60 °C.

Similarly, CFZ was found to be more susceptible to basic (95.2%) than to acidic (0.6%) degradation, consistent with the observations of Patil and Deshpande [53]. The spectral purity of the CFZ peak (Figure 6a) was 99.97%. No degradation peaks were detected for CFZ under acidic or basic stress conditions. The spectral purity of the CFZ peak (Figure 6b) was slightly lower (97.49%), possibly due to system noise dominating spectral variations, as the peak was relatively small. A degradation peak was detected in the chromatogram of the oxidized sample (Figure 6c) at an R_T_ of 1.626 min. This degradation product is highly polar and appears as an unretained peak. The spectral purity of the corresponding CFZ peak was 99.96%.

It can thus be concluded that the method demonstrated acceptable specificity, as no solvent, matrices, degradation product, or human skin (and receptor-fluid) interference was detected. The degradation products formed under the tested conditions were effectively detected and well separated from the two analytes of interest.

**Figure 7:**
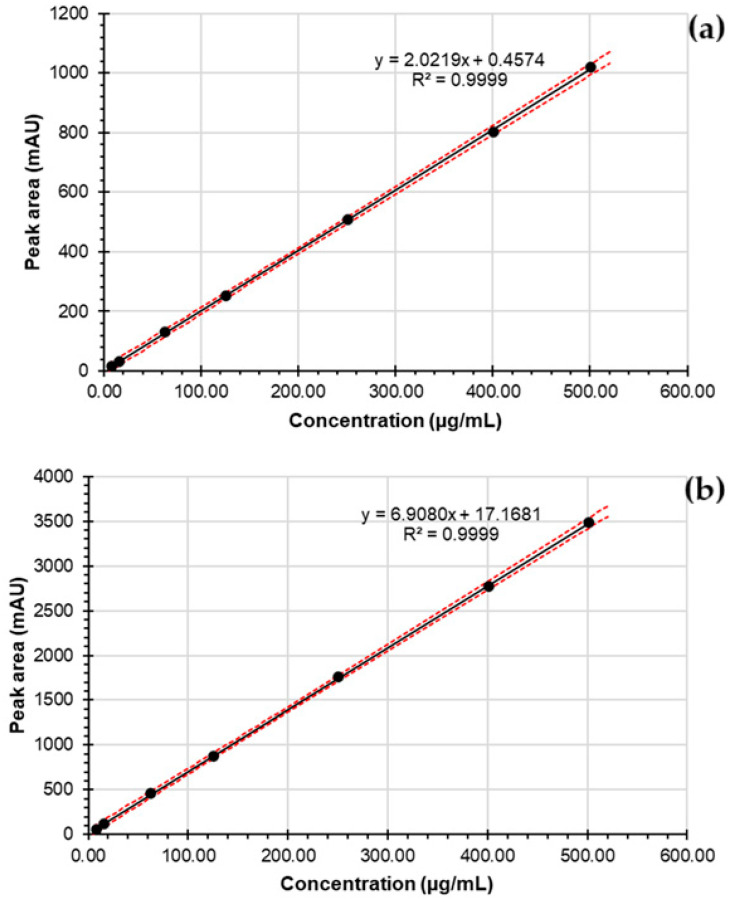
Calibration plots of (**a**) PZA and (**b**) CFZ across the working concentration range of 7.8–500.0 µg/mL. The 95% confidence intervals of the calibration lines are indicated by red dashed lines.

The external calibration curves (with 95% confidence intervals for the regression lines) for both drugs exhibited linear responses, as depicted in Figure 7. The calculated r^2^ supported strong linear relationships. ANOVA results indicated that the intercepts were statistically insignificant for both PZA and CFZ, with *p*-values > 0.05.

The LOQ of PZA was slightly lower than that of CFZ, which may be attributed to the lower response factor of PZA (2.05 mAU/µg·mL^−1^) compared to CFZ (7.23 mAU/µg·mL^−1^). The recovery of PZA ranged from 99.0% to 100.3% across the tested concentration levels, with a %RSD ≤ 0.98%, indicating excellent accuracy and precision. The recovery of CFZ ranged from 99.0% to 100.6%, with %RSD ≤ 0.61%, further supporting the method’s accuracy and precision.

Considering that the assay specification for the formulation requires that it contain no less than 90.0% and not more than 110.0% of the labeled amounts of PZA and CFZ, the %RSD required by the HPLC method at a 95% confidence interval (n = 3) was calculated to be no greater than 4.0% [54]. The intermediate precision results confirmed that the %RSD values for both individual sets and combined data were well within this limit (≤4.0%), demonstrating that the method is robust and produces consistent results across the working range and on different days. Table 4 summarizes the validation parameters and the results obtained.

**Table 4:** Validation parameters and results for PZA and CFZ quantitation.

Parameter	PZA				CFZ			
**Concentration range** (µg/mL)	7.8–500				7.8–500			
**Regression** Correlation coefficient (r^2^) Regression equation *p*-value for intercept	0.9999 y = 2.0219x + 0.4574 0.86				0.9999 y = 6.9080x + 17.1681 0.07			
**Limit of detection (LOD)** (µg/mL)	7.14				6.33			
**Limit of quantitation (LOQ)** (µg/mL)	21.62				19.18			
**Accuracy** Expressed as % recovery at ~500, 250, and 25 µg/mL	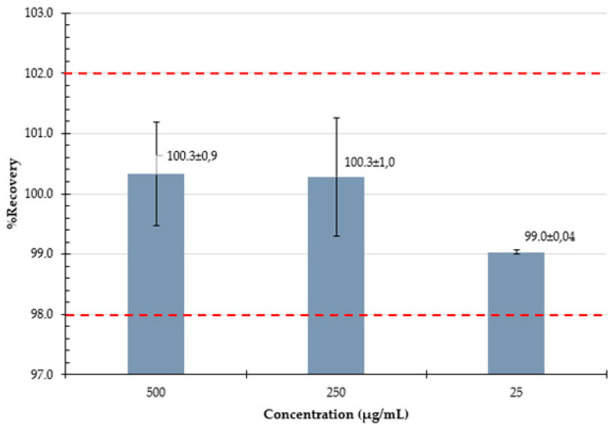				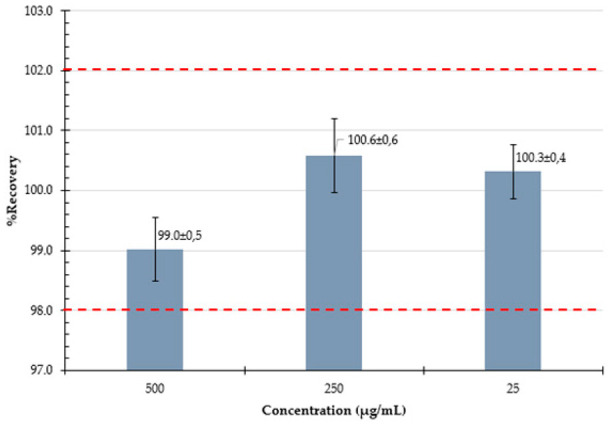			
**Intermediate precision** Determined at 3 levels, over 3 days		**Day 1**	**Day 2**	**Day 3**		**Day 1**	**Day 2**	**Day 3**
	**Level 1 ** **(~25 µg/mL)** $\bar{x}$ **:** **%RSD:**	99.3 98.3 99.0 98.4 99.5 99.0 99.0 0.37	99.3 99.1 99.3 99.1 98.8 98.9 99.1 0.21	99.1 98.9 99.3 99.1 98.8 98.8 99.0 0.22	**Level 1** **(~25 µg/mL)** $\bar{x}$ **:** **%RSD:**	100.0 100.2 100.0 99.8 99.6 99.4 99.8 0.28	99.7 100.0 99.8 100.2 102.0 100.5 100.4 0.85	100.3 101.2 100.7 100.7 100.5 101.0 100.7 0.32
	**Level 2** **(~250 µg/mL)** $\;\bar{x}$ **:** **%RSD:**	101.0 101.1 100.9 101.0 100.9 100.9 101.0 0.12	101.3 101.0 100.7 100.3 100.5 100.6 100.7 0.36	98.8 98.7 99.2 99.5 99.4 99.4 99.2 0.36	**Level 2** **(~250 µg/mL)** $\;\bar{x}$ **:** **%RSD:**	100.7 100.8 100.6 100.5 100.7 100.4 100.6 0.14	99.8 100.3 99.6 100.1 99.8 100.2 100.0 0.26	100.4 101.2 101.7 101.6 100.9 101.3 101.2 0.48
	**Level 3** **(~500 µg/mL)** $\bar{x}$ **:** **%RSD:**	100.3 100.7 100.9 100.6 101.1 101.2 100.8 0.33	100.8 100.9 101.2 100.7 100.7 100.9 100.9 0.19	99.1 99.3 99.5 99.4 99.3 99.4 99.3 0.16	**Level 3** **(~500 µg/mL)** $\;\bar{x}$ **:** **%RSD:**	98.6 99.9 100.1 99.7 99.8 99.5 99.6 0.53	98.2 98.8 98.3 98.6 98.8 98.8 98.6 0.26	99.1 98.8 98.7 98.5 99.0 99.0 98.8 0.24

### 4.3. Robustness of the Method—A Box–Behnken Experimental Design

A BBD was applied in the robustness study to investigate how deliberate changes to experimental parameters affect the instrument response and overall method performance. The relationships between the selected detection wavelength, mobile phase flow rate, and column temperature (each evaluated at three levels) and their impact on the responses of R_T_, peak tailing, and resolution between the PZA and CFZ peaks were investigated using 3D response surface plots.

Based on the chromatogram obtained (Figure 2) and pharmacopoeial requirements [45], the following desired chromatographic properties (i.e., critical quality attributes) were identified: (i) the desired R_T_ for PZA should range between 3 and 4 min to avoid potential interference from solvent or mobile phase peaks (1.7 to 2.5 min and 7.0 to 8.5 min), and that for CLF between 9 and 10 min to reduce potential interference from the slight baseline drift resulting from gradient changes (i.e., refractive index fluctuations in the eluent) [55]; (ii) the T_F_ should range between 1.0 and 1.5 [45], and (iii) the R_S_ between the PZA and CFZ peaks should not be less than 2.

The responses obtained following the experimental design are presented in Table 5. The data were subjected to statistical analysis and mathematical model fitting. A second-order polynomial model provided the best fit of all the responses. Table 6 presents a summary of the model-fit statistics for all dependent variables.

The values of the correlation coefficients and predicted correlation coefficients for the effects on the retention times of PZA and CFZ, PZA peak tailing, and peak R_S_ were found to be close (<0.2 difference), indicating acceptable prognostic efficiency of the experimental design for these response variables. It can thus be concluded that the retention time, tailing, and R_S_ of the PZA peak are influenced by the mobile phase flow rate, column temperature, and detection wavelength. Interestingly, while retention time is typically unaffected by the detection wavelength, variations in wavelength may alter the peak shape and intensity, influencing the detection of the peak apex and, consequently, the apparent retention time.

The r^2^ value describing the model fit for the T_F_ of the CFZ peak was low, indicating poor data fit. Furthermore, discrepancies between the r^2^ and adjusted r^2^ values suggested that the r^2^ value was artificially inflated due to the absence of statistically significant main and interaction effects at a 95% confidence level (*p* < 0.05) for all regression coefficients. This finding was confirmed by the ANOVA results, which showed no evidence that changes to the detection wavelength, flow rate, or column temperature—either individually or in combination—had a statistically significant effect on the tailing of the CFZ peak. This could be attributed to strong lipophilic interactions between the larger CFZ molecules and the stationary phase, which result in slower diffusion and greater mass-transfer resistance, making CFZ less sensitive to deliberate variations in the experimental parameters. PZA, on the other hand, is a smaller, hydrophilic molecule with weaker retention, eluting earlier in the gradient where changes in the flow rate, mobile phase composition, and column temperature are more pronounced. Consequently, its chromatographic behavior is more sensitive to changes in these independent variables, consistent with the behavior described by the van Deemter equation [56].

**Figure 8:**
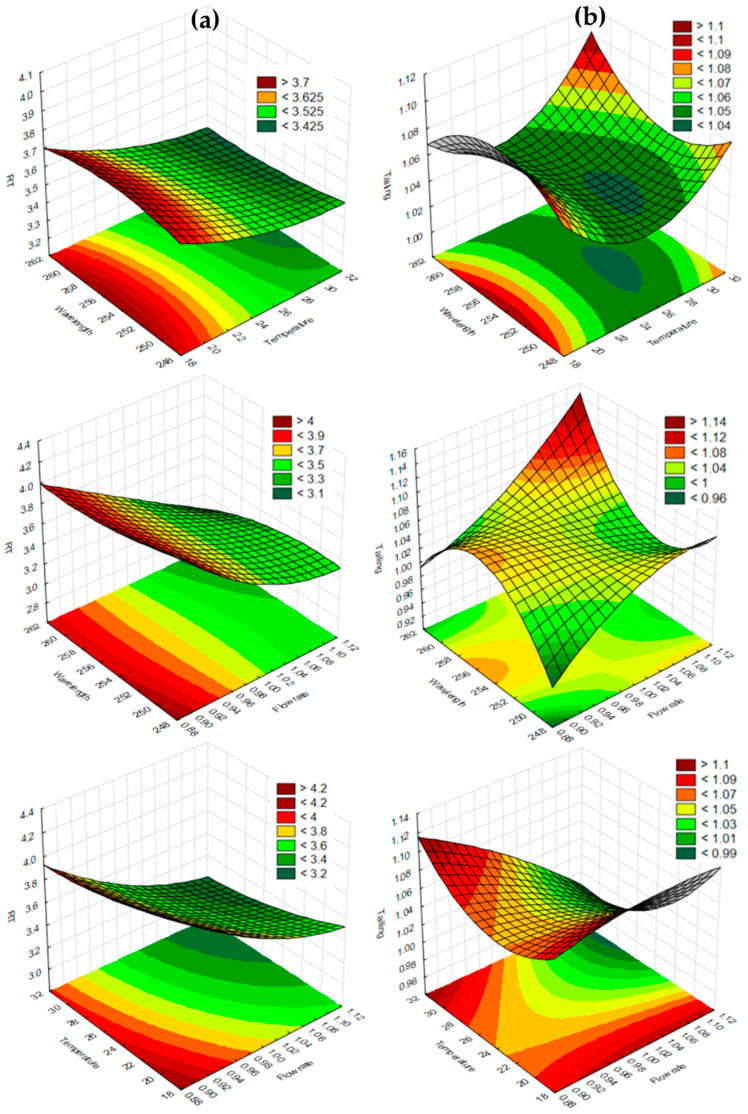

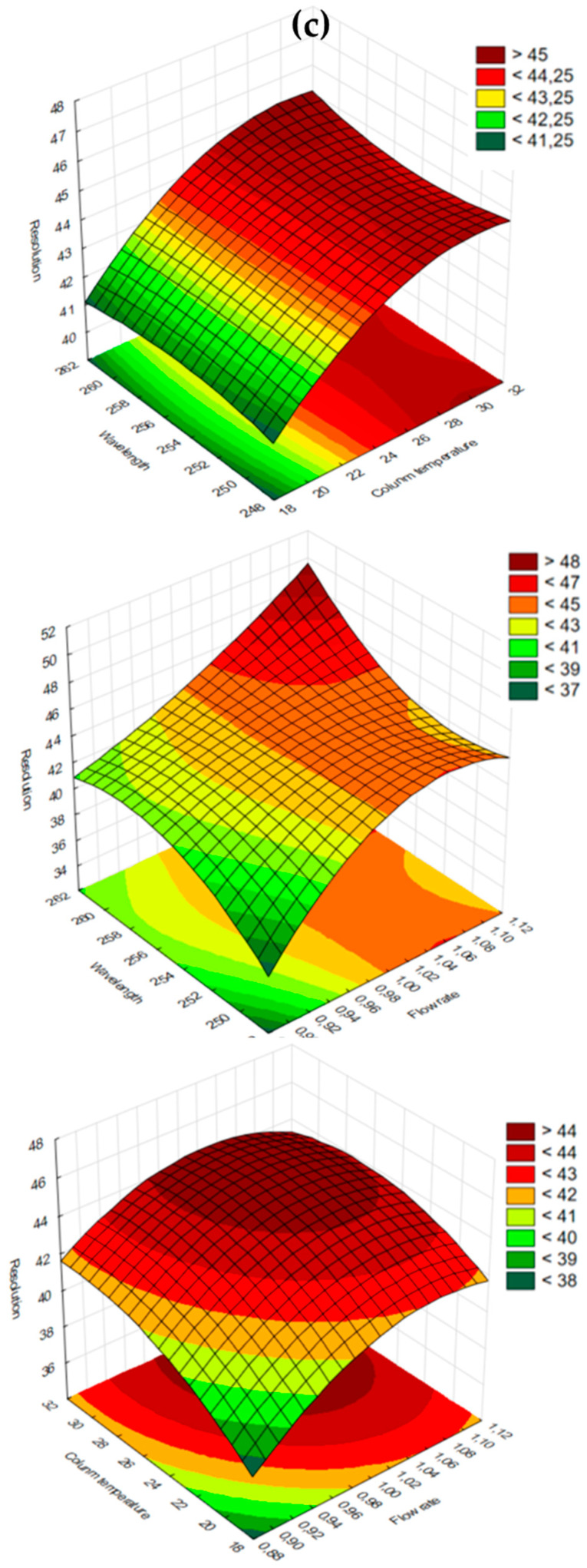
3D response surface plots illustrating the effects of the selected independent variables (i.e., detector wavelength, mobile phase flow rate, and column temperature) on the (**a**) retention time (R_T_) and (**b**) tailing factor of PZA and on the (**c**) resolution of PZA.

The 3D response surface plots illustrating the effects of the independent variables on the R_T_ of PZA (Figure 8a) were relatively flat with minimal curvature, indicating little interaction effects. Changes in the detection wavelength had little influence on the R_T_ of PZA. However, variations in the flow rate had the most significant impact on R_T_, with higher flow rates leading to shorter R_T_s. Column temperature also had a moderate effect, where increasing the temperature shortened the R_T_ of PZA, where increasing the column temperature resulted in a shorter R_T_, similar to literature findings [57]. The observed PZA R_T_ values (Table 5) ranged from 3.19 to 4.06 min across all experimental conditions, with an average R_T_ of 3.58 ± 0.14 min, which remained within the targeted R_T_ range (3–4 min). A similar trend was observed for CFZ, with R_T_ values ranging from 9.11 to 10.23 min, showing less variation (%RSD = 3.4%) compared to PZA (%RSD = 7.1%), supporting the robustness of the analytical method.

Interestingly, all three response-surface plots exhibit small but measurable curvature. The wavelength-temperature plot shows a gentle concave curvature with a central low region, suggesting optimal peak symmetry at a column temperature between 24 and 26 °C. The wavelength-flow rate plot displays a distinct saddle-shaped curvature [58] with a symmetry optimum (1 ≤ T_F_ ≤ 1.04) around the central flow rate (~1 mL/min). The temperature-flowrate plot displays a gentle valley with optimal symmetry at a 0.98 mL/min ≤ flowrate ≤ 1.12 mL/min at 32 °C ≤ column temperature ≤ 21 °C, confirming the optimized state of the new method. The symmetry of the CFZ peak (Table 5) remained acceptable as 1.0 ≤ T_F_ ≤ 1.1 under all the deliberate parameter variations.

**Figure 9:**
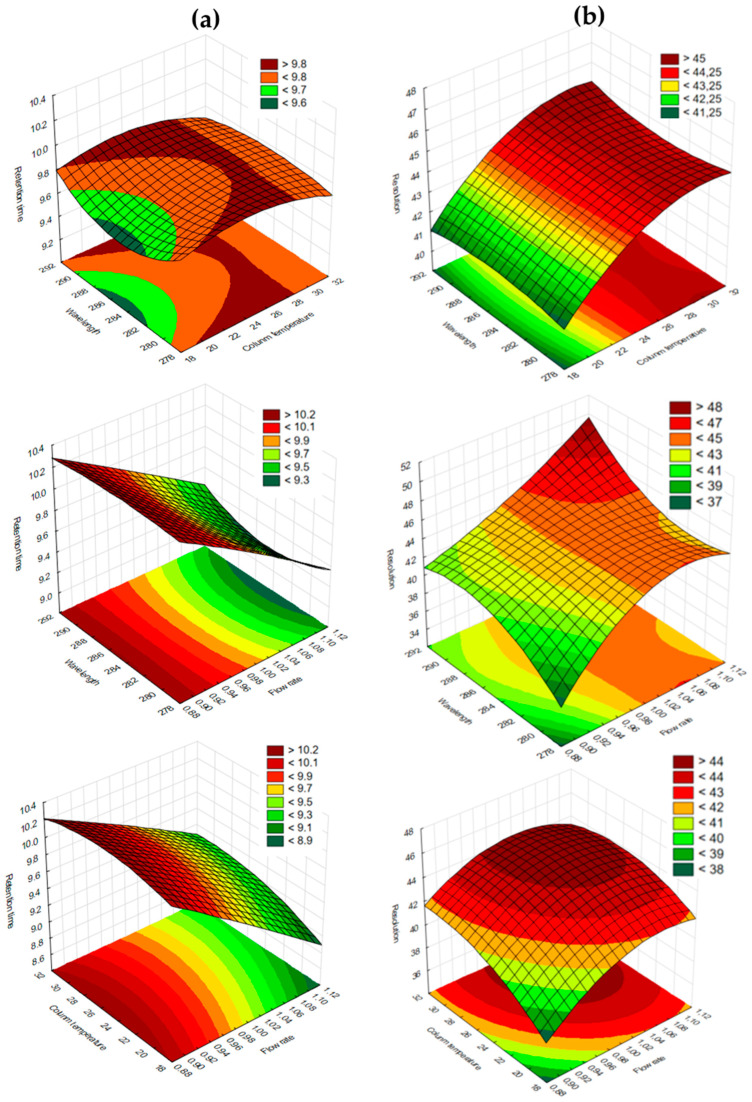
3D response surface plots demonstrating the effects of the designated independent variables (i.e., detector wavelength, mobile phase flow rate, and column temperature) on the (**a**) Retention time (R_T_) and (**b**) Resolution of CFZ.

The resolution surfaces shown in the 3D surface plots for PZA (Figure 8c) and CFZ (Figure 9b) portrayed strong curvature, indicating interaction effects among the independent variables. The flow rate was identified as the most critical factor influencing the resolution between PZA and CFZ, while the detection wavelength had the least effect, as expected, followed by column temperature. Across all combination scenarios, the R_S_ values ranged between 40 and 47. Although this represents an unusually high resolution, it may prompt the chromatographer to further optimize the method to achieve shorter elution times. However, doing so would require more rapid changes in the organic phase composition of the gradient due to the significantly large difference in the lipophilicity of the two drugs (PZA: Log P = −0.71 [24]; CFZ: Log P = 7.66 [31]). Preliminary tests showed that such adjustments resulted in the same transferability issues discussed earlier. Moreover, a slightly longer elution time was deemed advantageous to ensure the removal of nonpolar matrix components from the dermal formulation, thereby preventing increased backpressure and loss of column efficiency [59].

## 5. Conclusions

A rapid and reliable RP-DAD-HPLC method was developed to accurately quantify the solubility of CFZ and PZA in water, OLV, and selected mixtures of OLV + 1.5% (*v*/*v*) of a chosen essential oil (i.e., EUC; PPO; and TTO). The method may also be applied to quantify the amounts of PZA and CFZ in topical FDC emulsions prepared using the mentioned oil phases. Furthermore, this method can and has been used to measure dermal drug delivery from these emulsions. Therefore, it can be concluded that this versatile method is suitable not only for final quality control testing of formulated PZA and CFZ topical FDC emulsions, but also for pre-formulation studies (e.g., solubility analysis) and evaluation of drug release from the finished formulated product.

The use of a more conservative gradient program, combined with detection of both drugs at their optimal wavelengths using DAD, enhances method robustness by reducing the sensitivity to dwell-volume differences between chromatographic systems. The method was successfully validated in accordance with the ICH Q2 (R2) requirements.

## Figures and Tables

**Table 1:** Gradient program employed during the analysis.

Time (Minutes)	Mobile Phase A (% *v*/*v*)	Mobile Phase B (% *v*/*v*)	Comment
0–4	93.0–90.0	7.0–10.0	Linear gradient
4–6	90.0–50.0	10.0–50.0	Linear gradient
6–10	50.0	50.0	Isocratic
10–12	50.0–93.0	50.0–7.0	Linear gradient
12–15	93.0	7.0	Re-equilibration

**Table 2:** Levels of the independent variables used in the BBD optimization of the HPLC method.

Independent Variable	Levels		
	Low	Medium	High
*x*_1_ Wavelength for PZA (nm)	250	255	260
Wavelength for CFZ (nm)	280	285	290
*x*_2_ Flow rate (mL/min)	0.90	1.00	1.10
*x*_3_ Column temperature (°C)	20	25	30

**Table 3:** Results of forced PZA and CFZ degradation studies under the defined stress conditions.

Stress Conditions	Treatment of Samples	% Degradation Observed	
		PZA	CFZ
Acid degradation	0.1 N HCl for 3 h at 60 °C	0.4	0.6
Base degradation	0.1 N NaOH for 3 h at 60 °C	26.7	95.2
Oxidation	3% H_2_O_2_ for 3 h at 60 °C	0.0	82.8

**Table 5:** Robustness BBD experimental setup and results.

PZA							CFZ						
Run No.	Wave Length (nm)	Flow Rate (mL/min)	Column Temperature (°C)	R_T_ (min)	T_F_	R_S_	Run No.	Wave Length (nm)	Flow Rate (mL/min)	Column Temperature (°C)	R_T_ (min)	T_F_	R_S_
**1**	250	0.9	25	4.00	1.0	40	**1**	280	0.9	25	10.23	1.2	40
**2**	260	0.9	25	3.90	1.0	42	**2**	290	0.9	25	10.23	1.1	42
**3**	250	1.1	25	3.33	1.0	44	**3**	280	1.1	25	9.37	1.1	44
**4**	260	1.1	25	3.19	1.1	47	**4**	290	1.1	25	9.41	1.1	47
**5**	250	1.0	20	3.66	1.1	42	**5**	280	1.0	20	9.73	1.2	42
**6**	260	1.0	20	3.65	1.1	42	**6**	290	1.0	20	9.74	1.1	42
**7**	250	1.0	30	3.47	1.1	45	**7**	280	1.0	30	9.78	1.1	45
**8**	260	1.0	30	3.44	1.1	45	**8**	290	1.0	30	9.78	1.1	45
**9**	255	0.9	20	4.06	1.1	40	**9**	285	0.9	20	10.15	1.1	40
**10**	255	1.1	20	3.46	1.1	43	**10**	285	1.1	20	9.11	1.1	43
**11**	255	0.9	30	3.83	1.1	43	**11**	285	0.9	30	10.20	1.2	43
**12**	255	1.1	30	3.22	1.0	44	**12**	285	1.1	30	9.37	1.1	44
**13**	255	1.0	25	3.54	1.0	44	**13**	285	1.0	25	9.79	1.1	44
**14**	255	1.0	25	3.53	1.1	45	**14**	285	1.0	25	9.78	1.1	45
**15**	255	1.0	25	3.53	1.0	44	**15**	285	1.0	25	9.78	1.2	44
**16**	255	1.0	25	3.53	1.0	44	**16**	285	1.0	25	9.76	1.2	44
**Average:**				3.58	1.05	43		**Average:**			9.76	1.1	43
**Standard deviation:**				0.26	0.02	1.87		**Standard deviation:**			0.33	0.03	1.87
**Relative standard deviation (%):**				7.13	2.38	4.3		**Relative standard deviation (%):**			3.37	3.00	4.3

**Table 6:** Summary of the model-fit statistics for all dependent variables.

Dependent Variable	r^2^ Value	Adjusted r^2^ Value
R_T_ of PZA	0.99995	0.99975
T_F_ of PZA	0.95008	0.75404
R_S_ of PZA and CFZ	0.9921	0.97700
R_T_ of CFZ	0.99976	0.99879
T_F_ of CFZ	0.69829 *	0.00000 *
R_S_ of PZA and CFZ	0.9954	0.97700

* Poor fit—model not used.

## Data Availability

The raw data supporting the conclusions of this article will be made available by the authors upon request.

## Abbreviations

The following abbreviations are used in this manuscript:

AIDS	Acquired Immune Deficiency Syndrome
Adj. r^2^	Adjusted correlation coefficient
ANOVA	Analysis of Variance
AQbD	Analytical Quality-by-Design
AUC	Area Under the Curve
BBD	Box–Behnken Design
CFZ	Clofazimine
r^2^	Correlation coefficient
CTB	Cutaneous Tuberculosis
EUC	Eucalyptus oil
EPTB	Extrapulmonary Tuberculosis
FDC	Fixed-Dose Drug Combination
GMP	Good Manufacturing Practice
HIV	Human Immunodeficiency Virus
ICH	International Council for Harmonization
LOD	Limit of Detection
LOQ	Limit of Quantitation
OLV	Olive oil
PPO	Peppermint oil
PZA	Pyrazinamide
QbD	Quality-by-Design
%RSD	Relative Standard Deviation
R_S_	Resolution
RSM	Response Surface Methodology
T_R_	Retention Time
T_F_	Tailing Factor
TTO	Tea tree oil
TB	Tuberculosis
USP	United States Pharmacopoeia
WHO	World Health Organization
